# Anti-CD4-mediated selection of Treg *in vitro* – *in vitro* suppression does not predict *in vivo* capacity to prevent graft rejection

**DOI:** 10.1002/eji.200737562

**Published:** 2008-06

**Authors:** Vanessa Oliveira, Birgit Sawitzki, Stephanie Chapman, Christine Appelt, Inga Gebuhr, Joanna Wieckiewicz, Elaine Long, Kathryn J Wood

**Affiliations:** 1 Transplantation Research Immunology Group, Nuffield Department of Surgery, University of Oxford, John Radcliffe HospitalOxford, UK; 2 Institute of Medical ImmunologyCharité, Berlin, Germany

**Keywords:** Anti-CD4, IFN-γ, Transplantation, Treg

## Abstract

Regulatory T cells (Treg) have been shown to play a role in the prevention of autoimmune diseases and transplant rejection. Based on an established protocol known to generate alloantigen reactive Treg *in vivo*, we have developed a strategy for the *in vitro* selection of Treg. Stimulation of unfractionated CD4^+^ T cells from naive CBA.Ca (H2^k^) mice with C57BL/10 (H2^b^) splenocytes in the presence of an anti-CD4 antibody, YTS 177, resulted in the selection of Treg able to inhibit proliferation of naive T cells. *In vivo*, the cells were able to prevent rejection of 80% C57BL/10 skin grafts when co-transferred to CBA.Rag^–/–^ mice together with naive CD45RB^high^CD4^+^ cells. Purification of CD62L^+^CD25^+^CD4^+^ cells from the cultures enriched for cells with regulatory activity; as now 100% survival of C57BL/10 skin grafts was achieved. Furthermore, differentiation of Treg could be also achieved when using purified CD25^–^CD4^+^ naive T cells as a starting population. Interestingly, further *in vitro* expansion resulted in a partial loss of CD4^+^ cells expressing both CD62L and CD25 and abrogation of their regulatory activity *in vivo*. This study shows that alloantigen stimulation in the presence of anti-CD4 *in vitro* provides a simple and effective strategy to generate alloreactive Treg.

## Introduction

Naturally occurring regulatory T cells (nTreg) are produced in the thymus during normal T cell development 1 and are involved in the maintenance of self-tolerance 2–5 and development of tolerance to allogeneic transplants 6–8. Despite its broad spectrum of reactivity, the frequency of nTreg responding to foreign antigens is low and unable to provide protection against grafts mismatched for multiple major and minor histocompatibility antigens following transplantation 9, 10. On the contrary, Treg induced in response to antigen (iTreg) are generated from naive T cells under defined conditions *in vivo* and *in vitro* 2, 11–19. For therapeutic purposes, it may be possible to harness both the potential of nTreg as well as to generate T cells with regulatory activity to defined antigens, such as alloantigens, *ex vivo*. Repeated stimulation of unfractionated CD4^+^ cells in the presence of IL-10 or using a combination of immunosuppressive drugs induces the selection of Treg *in vitro*. These induced Treg are able to prevent colitis or central nervous system inflammation in animal models 20–22.

Pre-treatment of naive CBA mice with a non-depleting anti-CD4 mAb (YTS 177) and donor-specific (B10) transfusion enables the development of alloantigen-reactive CD4^+^ Treg, which render the recipient tolerant to a subsequent challenge with an allogeneic cardiac allograft 16, 23–25. The transplant provides a source of donor alloantigen indispensable for the persistence of the regulatory cells *in vivo* 26. In this study, we have addressed whether a similar strategy can be used *in vitro* to select and expand CD4^+^ Treg. We show that alloantigen stimulation of total CD4^+^ or CD25^–^CD4^+^ naive cells in the presence of a non-depleting anti-CD4 antibody (YTS177) drives the selection of a population of Treg that express CD25, CD62L, CCR7 and Foxp3 and are capable of suppressing the proliferation and cytokine expression of naive responder T cells, *in vitro*. Most importantly, these Treg are able to prevent the rejection of an allogeneic skin graft mediated by CD45RB^high^CD4^+^ cells when co-transferred into CBA.Rag^–/–^ recipients. Interestingly, expansion of the *in vitro* selected CD4^+^ cells with regulatory activity in the presence of IL-2 resulted in a loss of their capacity to prevent allograft rejection *in vivo*, although they retained their suppressive properties *in vitro*. This *in vitro* approach provides a complementary strategy to *in vivo* selection of Treg for controlling rejection and illustrates the tolerogenic potential of non-depleting anti-CD4 antibodies.

## Results

### Alloantigen stimulation in the presence of anti-CD4 antibody selects CD4^+^ cells with regulatory properties *in vitro* and *in vivo*

**Figure 1:**
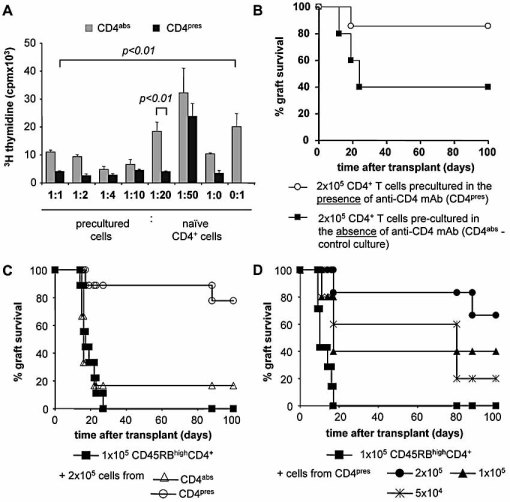
Influence of CD4^pres^ and CD4^abs^ on proliferation of naive T cells and skin graft rejection. (A) CD4^+^ cells precultured *in vitro* for 8 days with 5 μg/ml of anti-CD4 (YTS 177) (CD4^pres^) or without anti-CD4 antibody (CD4^abs^) were cultured with CD4^+^ responder T cells (2 × 10^5^) from naive CBA mice and irradiated allogeneic B10 splenocytes (5 × 10^5^). Proliferation was measured at day 5 by ^3^H-thymidine incorporation. Data are expressed as mean cpm + SD of triplicate cultures. The results shown are representative of four independent experiments. (B) CBA.Rag^–/–^ mice were reconstituted with 2 × 10^5^ CD4^pres^ (○; *n* = 7) or CD4^abs^ (▪, *n* = 5). The next day mice received an allogeneic skin graft from B10 mice. Results were pooled from two independent experiments. (C) CBA.Rag^–/–^ mice were reconstituted with 10^5^ CD45RB^high^CD4^+^ effector T cells from naive animals (control = MR mice; ▪, *n* = 9) or together with 2 × 10^5^ CD4^pres^ (○; *n* = 9) or CD4^abs^ (▵; *n* = 9). Results were pooled from five independent experiments. (D) CBA.Rag^–/–^ mice were reconstituted with 10^5^ CD45RB^high^CD4^+^ effector T cells from naive animals (control = MR mice; ▪, *n* = 7) or together with 2 × 10^5^ (•; *n* = 6), 10^5^ (▴; *n* = 5) or 5 × 10^4^ CD4^pres^ (*_gross;; *n* = 5).

Here, we asked whether *in vitro* blockade of CD4 signals to T cells at the time of alloantigen recognition, *i.e*. in the presence of allogeneic APC, would result in the selection of a population of CD4^+^ cells with suppressive properties. Purified, unfractionated CD4^+^ cells from naive CBA mice were cultured with irradiated, allogeneic splenocytes from B10 mice in the presence of 5 µg/mL anti-CD4. After 8 days in culture, their suppressive properties were investigated by coculturing the cells with naive, syngeneic CD4^+^ cells in the presence of allogeneic B10 splenocytes. As illustrated in Fig. 1A, CD4^+^ naive T cell responders failed to proliferate when cocultured with CD4^+^ cells precultured with anti-CD4 (CD4^pres^). At this ratio, CD4^+^ cells precultured without anti-CD4 (CD4^abs^) can also inhibit the proliferation of naive responders, demonstrating that CD4^pres^ or CD4^abs^ could suppress the proliferation of naive CD4^+^ responders *in vitro*. Differences between these two populations are detected at lower ratios (1:20) when CD4^abs^ loose their potential to suppress proliferation. *In vitro* suppression by CD4^pres^ may result from its ability to inhibit IL-2 and IFN-γ production by naive CD4^+^ cells (data not shown), however other mechanisms may be involved as CD4^pres^ express and secrete IL-10 and IFN-γ (data not shown).

Next, we tested whether CD4^pres^ have lost their potential to act as effector T cells, *i.e*. the ability to initiate rejection. CD4^pres^ (2 × 10^5^) or the same number of CD4^abs^ (control culture) were adoptively transferred into CBA.Rag^–/–^ mice and the following day a B10 skin allograft was transplanted. Six out of seven mice reconstituted with CD4^pres^ accepted their skin grafts (*n* = 7, median survival time (MST) >100 days, Fig. 1B), whereas three out of five mice reconstituted with CD4^abs^ rejected their skin grafts acutely (n = 5, MST = 24 days, *p*>0.05, Fig. 1B). Statistically, when compared to CBA.Rag^–/–^ mice reconstituted with CD45RB^high^CD4^+^ naive cells, both CD4^pres^ and CD4^abs^ have lost their potential to act as effector T cells *in vivo*. To assess the *in vivo* regulatory potential of the cultured CD4^+^ cells, CBA.Rag^–/–^ mice were reconstituted with 10^5^ CD45RB^high^CD4^+^ naive T cells and 2 × 10^5^ CD4^pres^ or CD4^abs^. CD4^pres^ prevented rejection in seven out of nine mice (*n* = 9, MST >100 days, *p*<0.0001 *vs.* MR), whereas most mice co-reconstituted with CD4^abs^ cells rejected the graft (*n* = 6, MST = 31 days, *p*<0.04 *vs.* 2 × 10^5^ CD4^pres^) (Fig. 1C). From these results we can conclude that the presence of anti-CD4 during *in vitro* culture enhances the regulatory capacity of CD4^+^ cells. Next, the minimum cell number to achieve *in vivo* regulation was determined. Therefore, varying cell numbers of CD4^pres^ were co-transferred with 10^5^ CD45RB^high^CD4^+^ naive T cells into CBA.Rag^–/–^ mice the day before transplantation of a B10 skin graft. Co-transfer of 2 × 10^5^ CD4^pres^ led to permanent acceptance of B10 skin grafts in four out of six mice (*n* = 6, MST = 94 days, *p*<0.05 *vs.* MR, Fig. 1D). Interestingly, transfer of 10^5^ or 5 × 10^4^ CD4^pres^ could still prevent rejection in a proportion of recipients (*n* = 5, MST = 49 days; *n* = 5, MST = 57.6 days, respectively. Fig. 1D). These results indicate that although both CD4^abs^ and CD4^pres^ are unable to induce graft rejection in the absence of an effector population, only CD4^pres^ can regulate skin graft rejection mediated by CD45RB^high^CD4^+^ cells *in vivo*.

### CD4^pres^ exhibit antigen specificity

**Figure 2:**
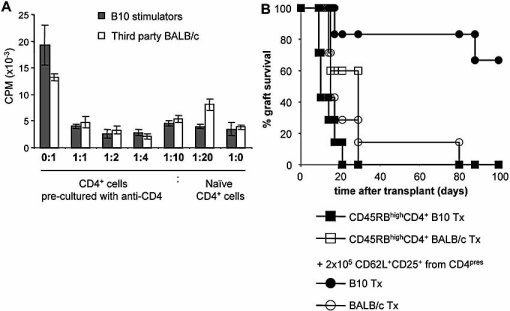
*In vitro* and *in vivo* third party responses of CD4^pres^. (A) CD4^pres^ were cultured at the indicated ratios with CD4^+^ responder T cells (2 × 10^5^) from naive CBA mice and irradiated allogeneic splenocytes either from B10 mice or from third party (BALB/c) mice (5 × 10^5^). Cells were cultured for 5 days and proliferation was measured by [^3^H]-thymidine incorporation. Data are expressed as mean cpm ± SD of triplicate cultures and are representative of three independent experiments. (B) CBA.Rag^–/–^ mice were reconstituted with 10^5^ CD45RB^high^CD4^+^ syngeneic T cells from naive animals (MR mice) or together with 2 × 10^5^ CD4^pres^. The next day, mice received an allogeneic skin graft from B10, or third party BALB/c, mice. MR mice acutely reject B10 (▪, *n* = 7) and BALB/c skin grafts (□, *n* = 5). Co-transfer of 2 × 10^5^ CD4^pres^ prevented rejection in four out of six mice receiving B10 skin grafts (•, *n* = 6) whereas none of the BALB/c skin grafts were accepted (○, *n* = 7). Results were pooled from two independent experiments.

It has been shown that regulatory T cells require activation through T cell receptor in order to manifest functional activity but thereafter are nonspecific, *i.e*. once these cells are activated 27, 28. Therefore, we wanted to test whether T cells with regulatory activity, CD4^pres^, could suppress the proliferation of naive T cells in an antigen specific or nonspecific fashion. CD4^pres^ were cultured with CD4^+^ cells from naive mice in the presence of either B10 or BALB/c allogeneic APC (Fig. 2A). Differential suppression, twofold, of responses to B10 or BALB/c stimulation was only detectable at very low ratios, 1:20 (Fig. 2A). These results showed that the response to third party antigen is not suppressed by CD4^pres^ to the same extent as the response to cognate antigen. Similar results were recently reported by Yamazaki *et al.* 29.

Next, we investigated the specificity of regulation by CD4^pres^ *in vivo*. CD4^pres^ precultured in the presence of B10 APC were unable to prevent the rejection of a BALB/c skin graft (*n* = 6, MST = 26.3 days, BALB/c skin grafts, *vs. n* = 6, MST = 94 days, B10 skin grafts; *p*<0.05, Fig. 2B). Rejection of third party BALB/c skin graft in mice co-transferred with CD4^pres^ and CD45RB^high^CD4^+^ cells was not significantly different from rejection in mice reconstituted with CD45RB^high^CD4^+^ cells alone (*n* = 5, MST = 23.4 days, *p* = 0.08, Fig. 2B). Hence, CD4^pres^ can suppress the proliferation of naive responders to third party stimulation *in vitro* but are unable to prevent the rejection of a third party skin graft *in vivo*.

### CD4^pres^ can be expanded further *in vitro* but lose regulatory activity

**Figure 3:**
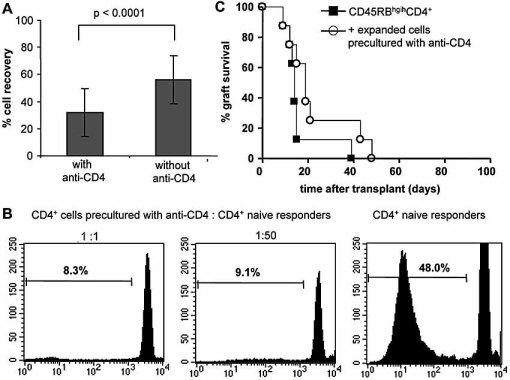
CD4^pres^ can be expanded *in vitro* but loose their potential to prevent graft rejection. (A) CD4^+^ cells from naive CBA mice (2 × 10^5^) were cultured for 8 days in the presence of anti-CD4 and 5 × 10^5^ allogeneic splenocytes from B10 mice. Percentage of cell recovery was calculated by dividing the number of viable CD4^+^ cells obtained after 8 days in culture by the numbers of CD4^+^ cells at the start of the culture. Data represent the mean ± SD of more than 40 independent experiments. (B) Cells were harvested and 5 × 10^4^ cells were restimulated for a further 7 days in the presence of 25 U/mL of human IL-2 (expanded CD4^pres^) and 5 × 10^5^ irradiated allogeneic splenocytes from B10 mice. After restimulation, cells were cultured with CFSE-stained CD4^+^ responder T cells (2 × 10^5^) from naive CBA mice and irradiated allogeneic B10 splenocytes (5 × 10^5^). CFSE staining was used to measure proliferation of the naive responder T cells after 7 days in culture. Numbers inside the histograms indicate the percentage of cells that went through, at least, one round of division. The results shown are representative of four independent experiments. (C) CBA.Rag^–/–^ mice were reconstituted with 10^5^ CD45RB^high^CD4^+^ effector T cells alone or together with 2 × 10^5^ IL-2 expanded CD4^pres^. The next day, mice received an allogeneic skin graft from B10 mice. MR mice acutely rejected B10 skin grafts (▪, *n* = 8). Co-transfer of 2 × 10^5^ IL-2 expanded CD4^pres^ did not prevent rejection of B10 skin grafts (○, *n* = 8).

Recently, studies by several groups have suggested that regulatory T cells can be expanded *in vitro* without loss of function using typical T cell growth factors such as IL-2 and IL-15 30, 31. The percentage of cell recovery of CD4^+^ cells after 8 days in culture in the presence of B10 APC and anti-CD4 is around 30% of the starting population (Fig. 3A). Therefore, we tested whether the population of CD4^pres^ could be expanded. After 8 days in culture with anti-CD4, CD4^+^ cells were restimulated with allogeneic splenocytes (B10) for a further 7 days in the presence of exogenous IL-2 (25 U/mL). Indeed, the addition of IL-2 resulted in an eightfold increase in the number of cells recovered from the cultures and the potential of the expanded CD4^pres^ to prevent proliferation of naive CD4^+^ cells *in vitro* was not diminished (Fig. 3B). Furthermore, suppression was still as potent at very low ratios such as 1:50 (8.3% proliferation at 1:50 *vs.* 9.1% at 1:1, Fig. 3B). In contrast to the non-expanded anti-CD4 Treg, as few as 4000 expanded CD4^pres^ were now sufficient to prevent proliferation of naive CD4^+^ cells.

Next, we investigated whether the expanded CD4^pres^ were able to prevent skin graft rejection *in vivo*. All mice reconstituted with CD45RB^high^CD4^+^ cells alone rejected their skin grafts acutely grafts (*n* = 8, MST = 14 days, Fig. 3C). Co-transfer of expanded CD4^pres^ could not prevent skin graft rejection although rejection occurred with a delayed kinetic (*n* = 8, MST = 11 days, *p*>0.05, Fig. 3C). These results indicate that although the expanded CD4^pres^ show a high regulatory potential *in vitro*, they were unable to prevent skin graft rejection *in vivo*.

### Cells with highest regulatory potential are present within the CD62L^+^CD25^+^ CD4^+^ subpopulation

**Figure 4:**
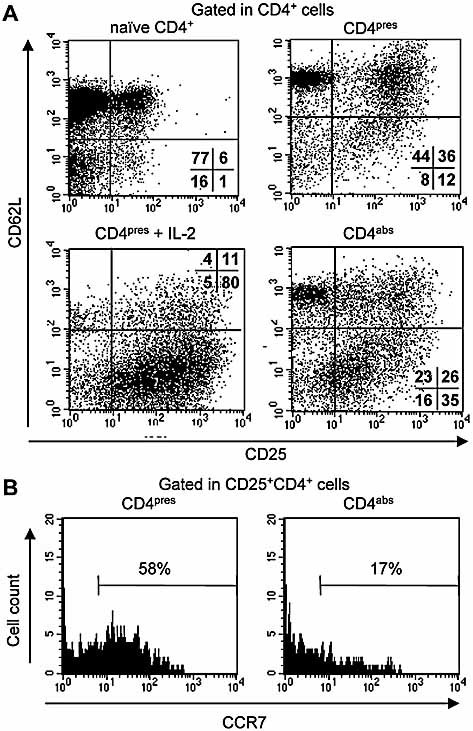
CD4^+^ cells cultured for 8 days in the presence of anti-CD4 exhibit a higher percentage of CD62L and CCR7 cells. (A) CD4^+^ cells from naive CBA mice and CD4^pres^ before and after IL-2-mediated expansion were stained with anti-CD4-PerCP, anti-CD62L-PE and anti-CD25-biotin and streptavidin-APC. Plots of CD62L *vs.* CD25 expression gated on CD4^+^ cells are shown. The data shown are representative of seven independent experiments. (B) The expression of CCR7 is shown gated in the CD4^+^CD25^+^ population. Numbers inside the histogram indicate the percentage of cells that stained positive for CD4, CD25 and CCR7. The data shown in (A) and (B) are representative of two independent experiments.

Lymphocyte extravasation from the blood circulation into high endothelial venules and subsequent entry into the secondary lymphoid organs involves a series of step-wise events, which involve CD62L and CCR7 32. The mAb, which block the interaction of CD62L with its ligands, prevent indefinite allograft survival and highlight the importance of this molecule for alloantigen tolerance 33. To understand the differences in regulatory potential *in vivo* between expanded and non-expanded CD4^pres^, the profile of cell-surface expression of both T cell populations was analysed. Cells were harvested after 8 days in culture (non-expanded CD4^pres^) or after a further 7 days in culture in the presence of IL-2 (expanded CD4^pres^) and stained with antibodies recognising CD62L and CD25. Of all non-expanded CD4^pres^, 36% expressed both CD25 and CD62L, whereas only 11% of the expanded population could be stained with both antibodies (Fig. 4A). These findings together with published studies from other groups 34–37 prompted us to investigate if this difference in CD62L expression could explain the results obtained *in vivo* (Fig. 1C and 3C). We analysed CD62L expression in CD25^+^CD4^+^ cells from CD4^pres^ and CD4^abs^ and found that 83 and 68% of cells, respectively, expressed CD62L. This surface marker is known to be necessary for cells to migrate in and out of lymph nodes. Following this observation, we decided to study CCR7 expression in CD25^+^CD4^+^ cells from CD4^pres^ and CD4^abs^ and found that, whereas 58% of CD25^+^CD4^+^ cells from CD4^pres^ express CCR7, this percentage decreased to 17% in CD25^+^CD4^+^ cells from CD4^abs^ (Fig. 4B).

**Figure 5:**
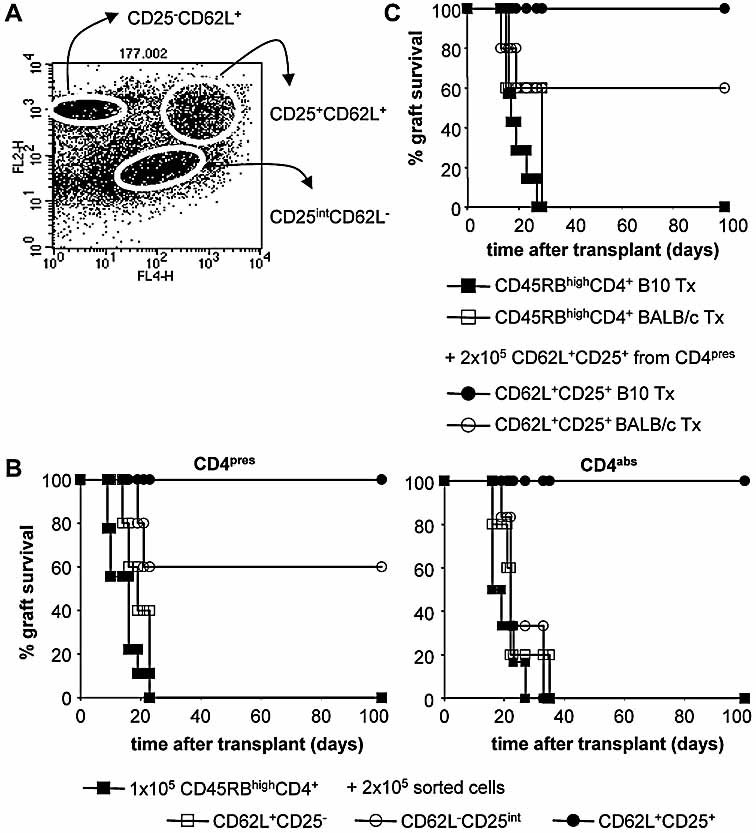
*In vivo* regulation is enriched within the CD62L^+^CD25^+^CD4^+^ subpopulation. (A) CD4^pres^ or CD4^abs^ were fractionated into three subpopulations based on surface expression of CD25 and CD62L. (B) CBA.Rag^–/–^ mice were reconstituted with 10^5^ CD45RB^high^CD4^+^ effector T cells alone (control = MR mice; ▪, *n* = 9) or together with 2 × 10^5^ of the following purified cell populations: CD62L^+^CD25^+^CD4^+^ cells from CD4^pres^ (•; *n* = 11), CD62L^–^CD25^int^CD4^+^ cells from CD4^pres^ (○; *n* = 5), CD62L^+^CD25^–^CD4^+^ cells from CD4^pres^ (□; *n* = 5), CD62L^+^CD25^+^CD4^+^ cells from CD4^abs^ (•; *n* = 5), CD62L^–^CD25^int^CD4^+^ from CD4^abs^ (○; *n* = 6) and CD62L^+^CD25^–^CD4^+^ cells from CD4^pres^ (□; *n* = 5). The next day the mice received an allogeneic skin graft from B10 mice. These results were pooled from four independent experiments. (C) CBA.Rag^–/–^ mice were reconstituted with 10^5^ CD45RB^high^CD4^+^ syngeneic T cells from naive animals (MR mice) or together with CD62L^+^CD25^+^CD4^+^ cells from CD4^pres^ cultures. The next day mice received an allogeneic skin graft from B10, or third party BALB/c, mice. MR mice acutely reject B10 (▪; *n* = 7) and BALB/c skin grafts (□; *n* = 5). Co-transfer of CD62L^+^CD25^+^CD4^+^ cells prevented rejection in five out of five mice receiving B10 skin grafts (•; *n* = 5) and three out of five of the mice receiving BALB/c skin grafts (○; *n* = 5). Results were pooled from three independent experiments.

Based on these observations, we investigated whether CD62L^+^CD25^+^CD4^+^ cells purified from CD4^pres^ or CD4^abs^ showed regulatory activity *in vivo*. Cells were separated by cell sorting into three cell populations according to their surface expression of CD25 and CD62L: CD62L^+^CD25^+^, CD62L^–^CD25^int^ and CD62L^+^CD25^–^ (Fig. 5A). CBA.Rag^–/–^ mice were reconstituted with 10^5^ CD45RB^high^CD4^+^ cells either alone or together with 2 × 10^5^ of each purified population from CD4^pres^ or CD4^abs^ (Fig. 5B). The following day, mice received a B10 skin allograft. *In vivo* regulatory properties resided in the CD62L^+^CD25^+^CD4^+^ population, regardless of their previous culture with (*n* = 11, MST >100 days, *p*<0.0001 *vs.* MR; CD4^pres^) or without anti-CD4 (*n* = 3, MST >100 days, *p*<0.001 *vs.* MR, CD4^abs^), as seen by long-term graft survival in all mice reconstituted with this population and CD45RB^high^CD4^+^ cells. In contrast, all mice receiving CD62L^+^CD25^–^CD4^+^ cells and CD45RB^high^CD4^+^ cells rejected their skin allografts with the same tempo as MR mice (Fig. 5B), irrespective of previous culture in the presence (*n* = 5, MST = 19.0 days, *p* = 0.2 *vs.* MR for CD4^pres^) or absence (*n* = 5, MST = 22 days, *p* = 0.4 *vs.* MR for CD4^abs^) of anti-CD4. In contrast, CD62L^–^CD25^int^CD4^+^ cells exhibited different regulatory capacity depending on previous culture with (*n* = 5, MST >100 days, *p*=0.055 *vs.* co-transfer of CD62L^+^CD25^+^ T cells) or without anti-CD4 (*n* = 6, MST = 22 days, *p*=0.1 *vs.* MR) (Fig. 5B). Thus, CD62L^–^CD25^int^CD4^+^ cells from CD4^pres^ cultures could prevent skin graft rejection mediated by CD45RB^high^CD4^+^ cells in three out of five mice. In contrast, none of the skin grafts survived permanently when CD62L^–^CD25^int^CD4^+^ cells from CD4^abs^ cultures were co-transferred with CD45RB^high^CD4^+^ cells. These data indicate that the anti-CD4 treatment results in phenotypic differences (higher frequency of CD62L and CCR7 positive cells) as well as functional changes (*in vivo* regulatory capacity of CD62L^–^CD25^int^CD4^+^ cells) of allo-stimulated CD4^+^ cells.

Interestingly, even 5 × 10^4^ CD62L^+^CD25^+^CD4^+^ cells from CD4^pres^ were capable of regulating a skin transplant (data not shown). In fact, 2 × 10^5^ CD62L^+^CD25^+^CD4^+^ cells were also able to regulate rejection of a skin transplant from BALB/c mice (third party) (*n* = 5, MST >100 days *vs.*MST >100 days, B10 skin grafts; *p*>0.05, Fig. 5C), indicating that this population can exert nonspecific regulation *in vivo*. Unfortunately, after expansion in IL-2, it was not possible to test the regulatory properties of the purified CD62L^+^CD25^+^CD4^+^ cells *in vivo* due to the low numbers of cells recovered from these cultures. Taken together, these results indicate that regulatory properties reside mainly in CD62L^+^CD25^+^CD4^+^ cells purified either from CD4^pres^ or CD4^pres^ but also in CD62L^–^CD25^int^CD4^+^ cells of CD4^pres^ cultures.

### The highest percentage of FoxP3^+^ cells can be found in CD62L^+^CD25^+^CD4^+^ and CCR7^+^CD25^+^CD4^+^ cells

**Figure 6:**
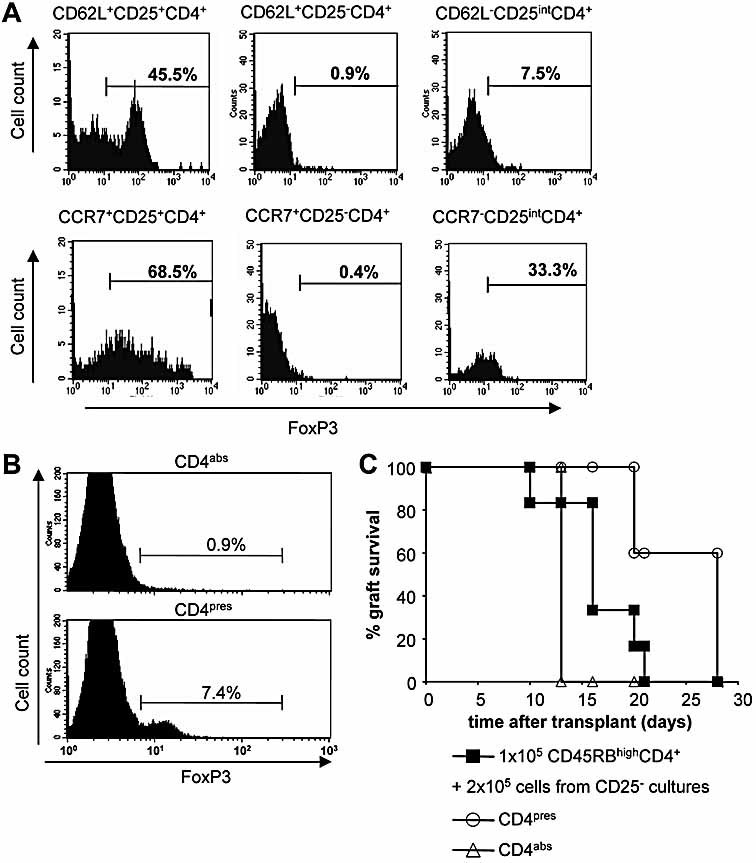
Foxp3 expression and *in vivo* regulatory capacity when using CD25^–^CD4^+^ naive T cells as responders. (A) CD4^pres^ were stained with anti-CD4-FITC, anti-CD62L-PE or anti-CCR7-PE, anti-CD25-biotin and streptavidin-CyChrome and FoxP3-APC. The expression of FoxP3 is shown after gating in the indicated subpopulations from CD4^pres^. Numbers inside the histogram indicate the percentage of cells that stained positive for CD4, CD25, CD62L or CCR7 and FoxP3. The data shown are representative of two independent experiments. (B) Foxp3 expression gated on CD4^+^CD25^+^ cells of CD4^pres^ and CD4^abs^ cultures of CD25^–^CD4^+^ naive T cells. (C) CBA.Rag^–/–^ mice were reconstituted with 10^5^ CD45RB^high^CD4^+^ effector T cells from naive animals (control = MR mice) or together with 2 × 10^5^ CD4^pres^ or CD4^abs^. MR mice acutely reject B10 skin grafts (▪, *n* = 6; MST = 16.5 ± 3.5 days). Co-transfer of 2 × 10^5^ CD4^pres^ prolonged skin graft survival (○, *n* = 5; MST = 24.8 ± 3.9 days) whereas co-transfer of 2 × 10^5^ CD4^abs^ resulted in accelerated rejection (▵, *n* = 3; MST = 13.0 ± 0.0 days).

FoxP3 expression has been shown to characterize nTreg 38, 39 and to be up-regulated in transgenic T cells cultured with bone marrow dendritic cells and specific antigen 14. For these reasons we studied FoxP3 expression in subpopulations sorted from CD4^pres^. As shown in Fig. 6A, CD62L^+^CD25^+^CD4^+^ cells exhibit higher levels of FoxP3 (45.5%) than CD62L^–^CD25^int^CD4^+^ (7.5%) and CD62L^+^CD25^–^CD4^+^ cells (0.9%) cells. These results are in accordance with the regulatory potential found *in vivo* for each of these cell populations (Fig. 5B). When studying FoxP3 expression in CCR7^+^CD25^+^CD4^+^, CCR7^–^CD25^int^CD4^+^ and CCR7^+^CD25^–^CD4^+^ cells from CD4^pres^ we found similar results (68.4, 33.3 and 0.4%, respectively, Fig. 6A). The same pattern was found for subpopulations sorted from CD4^abs^ (data not shown). Taken together, these results indicate that FoxP3^+^ cells reside mainly in the CD62L^+^CD25^+^CD4^+^ or CCR7^+^CD25^+^CD4^+^ subpopulation. Despite this, CD62L^–^CD25^int^CD4^+^ cells are also able to regulate rejection of a skin allograft, which may give us the indication that CD25 expression and other factors are more important for *in vivo* regulation than CD62L. This assumption is further supported by our observation that the CD62L^+^CD25^+^CD4^+^ subpopulation can be found in the draining lymph nodes and in the skin allograft and does not prevent *in vivo* migration or proliferation of CD45RB^high^CD4^+^ cells (data not shown).

Induced regulatory T cells can be differentiated from CD25^–^CD4^+^ naive T cells.

We have also performed experiments using CD25^–^CD4^+^ cells from naive CBA mice as responders in our anti-CD4 mAb-treated mixed lymphocyte culture. Using our established protocol we were able to generate 7.4% CD62L^+^CD25^+^Foxp3^+^ cells after 8 days of culture (Fig. 6B). In contrast, in control cultures 0.9% of the CD25^–^CD4^+^ cells became CD62L^+^CD25^+^Foxp3^+^. We also tested their *in vivo* regulatory capacity. CBA.Rag^–/–^ mice were reconstituted with 10^5^ CD45RB^high^CD4^+^ cells either alone or together with 2 × 10^5^ cells from unfractionated CD4^abs^ or CD4^pres^ cultures set up using CD25^–^CD4^+^ T cells. The following day, mice received a B10 skin graft. Co-transfer of CD4^pre^s cells resulted in a prolongation of skin graft survival (MST ± SD: 24.8 ± 3.9 days, *p*=0.009) in comparison to recipients reconstituted with CD45RB^high^CD4^+^ cells only (MST ± SD: 16.5 ± 3.5 days, Fig. 6C). Whereas co-transfer of CD4^abs^ cells resulted in accelerated skin graft rejection (MST ± SD: 13 ± 0 days). These data clearly indicate that anti-CD4 mAb treatment during mixed lymphocyte cultures facilitates both a selective expansion of initial natural CD4^+^CD25^+^Foxp3^+^ regulatory T cells but also a differentiation of alloantigen-specific induced regulatory T cells, which makes the anti-CD4 mAb treatment-based protocol even more attractive for future clinical applications.

## Discussion

Based on an established *in vivo* protocol known to select alloantigen-reactive CD25^+^CD4^+^ regulatory T cells *in vivo* 16, 23, we investigated whether a similar strategy would result in the selection of T cells with regulatory potential *in vitro*. Such an *in vitro* approach is attractive, as the *ex vivo* generated regulatory cells could be used throughout the post-transplant course to enhance or sustain the unresponsive state achieved by the *in vivo* therapy. In this system, mouse CD4^+^ cells were purified and cultured for 8 days with alloantigen in the form of allogeneic APC and in the presence of a non-depleting anti-CD4 mAb. This *in vitro* combination led to the selection, of CD4^+^ cells, designated CD4^pres^, secreting high amounts of IL-10 and IFN-γ (data not shown) that are able to suppress the proliferation of naive syngeneic T cells (Fig. 1A). More importantly, CD4^pres^ are able to prevent rejection mediated by co-transfer of naive T cells (Fig. 1B and C). This (lack of rejection) cannot be explained by an activation induced anergy of CD4^pres^, as rejection occurs in CBA.Rag^–/–^ reconstituted with CD45RB^high^CD4^+^ naive T cells only. Active regulation is required to prevent rejection mediated by naive T cells. We believe that culture of naive CD4^+^ cells with alloantigen in the presence of anti-CD4 results in an enrichment and selection of Treg.

It is generally thought that protocols generating Treg for clinical application need to have the capacity to produce large numbers of cells that retain their regulatory activity *in vivo*. When we expanded CD4^pres^ further *in vitro*, we found that while the expanded population retained potent suppressor function *in vitro*, they completely lost regulatory capacity *in vivo* (Fig. 3B and C). This loss of functional activity *in vivo* may result from the dramatic decrease in the percentage of cells co-expressing CD25 and CD62L and functional changes of the CD62L^–^CD25^int^CD4^+^ population after expansion with IL-2 (Fig. 4A). *In vivo* regulatory potential resided mainly in the CD62L^+^CD25^+^CD4^+^ subpopulation purified from either the CD4^pres^ or CD4^abs^ cultures, but there was also evidence for regulatory activity in CD62L^–^CD25^int^CD4^+^ of CD4^pres^ cultures (Fig. 5B). The percentage of CD62L^+^ or CCR7^+^ cells in the CD25^+^CD4^+^ population is higher in cultures in the presence of anti-CD4 (Fig. 4B) and the anti-CD4 treatment results in functional changes of CD62L^–^CD25^int^CD4^+^cells, which again demonstrates the important role of this antibody for the *in vitro* enrichment and selection of Treg.

CD62L^+^CD25^+^CD4^+^ cells from CD4^pres^ cultures exhibited higher percentages of FoxP3 expression than either CD62L^+^CD25^–^CD4^+^ or CD62L^+^CD25^int^CD4^+^ cells (Fig. 6A). The percentage of FoxP3^+^ cells found in CD62L^–^CD25^int^CD4^+^ varied between experiments, but it was always lower than that found in CD62L^+^CD25^+^CD4^+^ cells. *In vivo*, CD62L^+^CD25^+^CD4^+^ cells from CD4^pres^ cultures were detected both in the draining lymph node and in the skin graft 7 days after transplantation, but were not detected in the spleen of reconstituted CBA.Rag^–/–^ mice (data not shown). These results are in accordance with observations from other groups showing that CD62L^+^CD25^+^CD4^+^ cells expressing CCR7 are able to delay diabetes transfer in NOD mice 36. Expression of CD62L was also found to be important for the localization of regulatory cells into secondary lymphoid organs in models of cardiac transplantation 33 and graft-*versus-*host disease 40. Furthermore, recently it was shown that CCR7 is required for the *in vivo* function of CD25^+^CD4^+^ regulatory T cells 41. Thus, we can speculate that CD62L and CCR7 expression is required for regulatory cells to migrate into the lymph nodes and regulate the priming of naive T cells, but other factors induced by the anti-CD4 treatment are also important.

To increase the amount of antigen-specific regulatory T cells for *in vivo* cell therapy, a protocol aimed on generating Treg should also enable differentiation of induced Treg from CD25^–^CD4^+^ naive precursors. It has been shown that polyclonal stimulation of naive T cells in the presence of TGF-β results in the differentiation of induced Treg 42, 43. Our results demonstrate that also stimulation of CD25^–^CD4^+^ naive T cells with alloantigen in the presence of the non-depleting anti-CD4 antibody YTS177 enables differentiation of CD25^+^Foxp3^+^ induced Treg with high regulatory potential *in vivo*.

The ultimate challenge for our in vitro generation protocol would be to test the regulatory capacity of CD4_pres_ cells upon transfer into fully immunocompetent recipients. Indeed, we are currently performing experiments in which we transfer CD4_pres_ cells into otherwise untreated naïve C57BL/6 mice receiving an allograft.

In situations characterised by high responder frequencies such as stringent strain combinations and most likely in clinical transplantation we need powerful protocols for *in vitro* generation and differentiation of antigen specific regulatory T cells. Our protocol presented here may be one approach to generate such potent Tregs.

Recently, Golshayan *et al.* 44 have presented a protocol of selecting and expanding Treg with indirect allospecificity for a single MHC class I antigen (H2K^b^) from a pool of naturally occurring Treg using H2K^b^ peptide-pulsed immature dendritic cells. The expanded Treg lines could regulate indirect alloresponses of effector CD4^+^ cells *in vivo*. In contrast, the expanded Treg could not inhibit direct pathway alloresponses, such that C57BL/6 skin transplants were rejected with the same kinetic as in control mice 44. These data indicate that Treg with a broader spectrum of alloreactivty is most likely required to control rejection of a fully allogeneic graft. The protocol for generating Treg reported here is aimed at selecting Treg controlling direct pathway immune responses and therefore complements the findings made by Golshayan *et al.* 44.

The underlying mechanism by which anti-CD4 antibody treatment induces the enrichment/selection of Treg *in vitro* remains to be investigated. CD4 is an important accessory and costimulatory molecule that augments the signal received by the TCR complex and helps in the activation of the T cells 45. Blockade of this interaction using anti-CD4 antibodies increases the threshold of activation through the TCR, so that, under these conditions, only cells with high affinity have the capacity to be activated and proliferate 46. These cells with high affinity/specificity TCR could be identical with cells that manifest regulatory activity in this study. Indeed, autoreactive CD25^+^CD4^+^ regulatory cells can be generated in the thymus by interactions with a single self-peptide. Selection of these cells appears to require a TCR with high affinity for a self peptide as thymocytes with low-affinity TCR do not undergo selection into this pathway 47. Furthermore, reduced or blocking levels of CD4 expression, can convert T cell responses from stimulatory to inhibitory 48. We can speculate that using the protocol described here, T cell activation has purposefully become more difficult and only a small percentage of cells can undergo activation and proliferation (regulatory population), while cells that do not receive any signals eventually die by apoptosis (these include the effector population). Indeed, in our previous studies we could show that anti-CD4 mAb-treated allo-reactive T cells are characterised by a relative resistance to activation-induced cell death (AICD) 49. Thus, we can conclude that anti-CD4 mAb treatment of allo-reactive T cells facilitates the expansion and differentiation of antigen.specific Treg, which are characterised by a relative resistance to AICD, whereas effector T cells dye by apoptosis. Alternatively, the anti-CD4 antibody may directly deliver a negative signal into the T cells 50 and thereby drive the selection of a population of Treg instead of selecting it from a pre-existing pool. Interestingly, a recent study performed by McFadden *et al.* 51 has revealed that stimulation with the CD4 ligand IL-16 results in enhanced migration and *de novo* induction of a regulatory population of CD25^+^CTLA4^+^Foxp3^+^ cells. It is likely that TCR affinity and CD4 expression levels at the cell surface both play equal roles in our protocol. Following this line of thinking, we believe that it may be possible to develop protocols, targeting one or more accessory or costimulatory molecules 11, to generate stringent culture conditions in which only a specific T cell population (regulatory population) can be selected 20.

Our findings may help to understand the mechanisms underlying the function of Treg *in vitro* and *in vivo*, which will enable its use the cells for therapeutic strategies *in vivo*. This protocol, stimulating T cells with alloantigen-presenting cells in the presence of anti-CD4, provides a powerful, simple and fast method of obtaining cells *ex vivo* with strong regulatory potential but also illustrates the immense tolerogenic and therapeutic potential of non-depleting anti-CD4 antibodies.

## Materials and methods

### Mice

CBA.Ca (CBA, H2^k^), C57BL/10 (B10, H2^b^), BALB/c (H2^d^), CD52-transgenic CP1-CBA.Ca (H-2^k^), and CBA.Rag 1^–/–^ (H2^k^) were bred and housed in the Biomedical Services facility at the John Radcliffe Hospital (Oxford, UK) in accordance with the Animals (Scientific Procedure) Act 1986 of the UK. Sex-matched mice aged 6–12 weeks were used in all experiments.

### Reagents and monoclonal antibodies

The following reagents were used for *in vitro* assays and flow cytometry. The hybridomas YTS 169.4.2 (anti-CD8) and YTS 177.9 (“YTS 177”, anti-CD4) were kindly provided by Professor Herman Waldmann (Sir William Dunn School of Pathology, Oxford, UK). TIB120 (anti-class II) was obtained from American Type Culture Collection (ATCC) Manassas, VA). RM4–5 (anti-CD4)-cychrome, 16A (anti-CD45RB)-PE, MEL-14 (anti-CD62L)-PE, 7D4 (anti-CD25)-biotin and streptavidin-allophycocyanin were from PharMingen (San Diego, CA). 4B12 (anti-CCR7)-PE and FJK-16s (anti-FoxP3)-APC were from eBioscience (San Diego, CA). CFSE was from Molecular Probes (OR).

### Cell preparation and purification

#### Purification of T cell subpopulations

Single-cell suspensions from spleen and lymph nodes of naive CBA mice were prepared by forcing the organs through a 70-µm nylon mesh. After erythrocyte removal by hypotonic lysis, cell suspensions were incubated with YTS169 (anti-CD8, 200 µg/mL) and TIB120 (anti-class II, 100 µg/mL) at 2 × 10^8^ cells/mL. Cell suspension was incubated with sheep anti-rat-coated Dynabeads (Dynal A.S., Oslo, Norway). CD8^–^ and MHC class II^–^ cells were isolated by magnetic separation, the CD4^+^-enriched population resuspended and stained for CD4 and CD45RB. CD45RB^high^CD4^+^ cells with 99% purity were obtained by cell sorting using a FACSVantage (Becton Dickinson, San Jose, CA).

#### Cell culture

RPMI 1640 culture medium was supplemented with 10% FBS, 2 mM L-Glutamine, 0.5 mM 2-mercaptoethanol, and 100 U/mL each penicillin, streptomycin and kanamycin. Irradiated (3600 rad) allogeneic total splenocytes from B10 mice were used as APC (B10 APC). Purified CD4^+^ cells (mouse CD4^+^ isolation kit Miltenyi Biotec, Bergisch-Gladbach, Germany) from naive CBA mice were cultured at 2 × 10^5^ cells/well in the presence (CD4^pres^) or absence (CD4^abs^) of 5 µg/mL of YTS 177 anti-CD4 mAb together with 5 × 10^5^ APC. Cells were cultured for 8 days, harvested, debris separated from live cells by gradient centrifugation using lymphoprep medium (Axis-Shield PoC AS, Oslo, Norway) and used in a regulation assay. In some experiments, CD4^+^ cells precultured with anti-CD4 (5 × 10^4^) were restimulated with 5 × 10^5^ APC in the presence of 25 U/mL human IL-2 (Roche) (expanded CD4^pres^). Proliferation of naive responders was assessed by [^3^H]thymidine (0.5 µCi) incorporation or CFSE dilution after 5 or 7 days in culture, respectively.

### Cell reconstitution and skin transplantation

CBA.Rag 1^–/–^ mice were reconstituted intravenously with syngeneic fractionated T cells. CD45RB^high^CD4^+^ cells (10^5^) purified from naive CBA mice were used routinely to elicit rejection of an allogeneic skin graft 16. The day after reconstitution, all mice received a B10 skin graft as previously described 16. Graft rejection was defined as complete destruction of the skin graft.

### Flow cytometric analysis

Cell cultures were incubated with the appropriate antibodies. All incubations were carried out for 20 min at 4ºC. Data were acquired using a FACSort (Becton Dickinson, San Jose, CA) and analysed using CellQuest software package (Becton Dickinson). Intracellular staining for FoxP3 studies was performed according to the manufacturer's instructions.

### Statistics

The Log Rank Method was used to compare allograft survival between groups using GraphPad Prism version 3.00 for Windows (GraphPad Software, San Diego, CA).

The data were analysed using a two-tailed unpaired Student's *t*-test. Results are given as the mean ± SD. *p*-values of <0.05 were considered significant.

## Abbreviations

MST: median survival time
nTreg: naturally occurring regulatory T cell
